# Enzymatic synthesis of some sugar-lauric acid esters by lipase from *Candida antarctica* and their functionalities as emulsifiers and antibacterial agents

**DOI:** 10.1016/j.fochx.2025.102383

**Published:** 2025-03-17

**Authors:** Kangzi Ren, Guilin Chen, Ziyi Zhang, Zhao Long, Bo Zhou, Wenfang Han, Qinlu Lin

**Affiliations:** a College of Food Science and Engineering, Central South University of Forestry and Technology, Changsha, Hunan 410004, China; b National Engineering Research Center of Rice and Byproduct Deep Processing, 498 South Shaoshan Road, Hunan 419994, China

**Keywords:** Sugar ester, Lauric acid, Lipase-catalyzed synthesis, Emulsifiers, Foam, Antibacterial

## Abstract

To investigate the regioselectivity of lipase-catalyzed synthesis of important sugar-laurate esters and their functionalities as emulsifiers and antimicrobial agents, glucose, galactose, mannose, maltose and trehalose were used. 6-*O*-lauryl glucose (Glu-L)*,* 6-*O*-lauryl galactose (Gal-L)*,* 6-*O*-lauryl mannose (Man-L)*,* 6′-*O*-lauryl maltose (Mal-L) *and* 6-*O*-lauryl trehalose*/*6’-*O*-lauryl trehalose (Tre-L) were synthesized using lipase from *Candida antarctica*. The Glu-L and Man-L achieved the highest yields (65.49 % and 58.16 %, respectively). The synthesized esters produced smaller-particle-size (1.9–3.1 μm), but less stable emulsions than the commercial sucrose esters (4.2–8.1 μm). The zeta-potential data revealed Man-L and Gal-L had higher surface coverage than the Glu-L on the oil droplets, while the Mal-L and Tre-L had similar surface coverage. The Man-L, Mal-L and Tre-L demonstrated the superior foamability and foam stability. The Gal-L, Man-L, Mal-L, and Tre-L inhibited *E.coli* 12024 (MIC 1–4 mg/mL), while only Man-L, Mal-L, and Tre-L inhibited *B.subtilis* 5009 (MIC 0.5–2.0 mg/mL).

## Highlights

• Five types of sugar monolaurate esters were synthesized by enzymatic method.
• The acylation positions were either at 6- or 6′- position of the sugar structure.
• The five esters formed smaller size but less stable emulsions than sucrose esters.
• Mannose-, maltose- and trehalose- esters excelled in fomability and foam stability.
• Galactose-, mannose-, maltose- and trehalose esters showed antimicrobial efficacy.

## 1 Introduction

Sugar-fatty acid esters (SFAE) are amphiphilic molecules that consist of hydrophilic groups (sugar molecules) and hydrophobic groups (fatty acids) that are linked by ester bond. These compounds are biodegradable, non-toxic, odorless and non-irritant to skin (Ferrer, Cruces, Bernabé, Ballesteros, & Plou, 1999). SFAE can be used as emulsifiers in food, pharmaceutical, cosmetic and detergent products. Beyond their emulsifying capabilities, SFAE were reported to have antimicrobial properties against several bacteria and fungi (AlFindee et al., 2018). SFAE can be synthesized by chemical methods and enzymatic methods. The currently commercialized SFAE (primarily sucrose esters) are chemically synthesized. Enzymatic synthesis methods require lower temperatures and fewer steps (Teng et al., 2021). Thus, the synthesis method may consume less energy and can be regarded as more environmentally friendly. The sustainability of food and food ingredients has been reported to be a driving factor in consumers' food choice preferences (Asioli et al., 2017). From the industrial point of view, the enzymatic process yields higher purities of products because of the enzyme's regioselectivity (Teng et al., 2021), which may simplify the purification processes. Therefore, it is worthwhile to explore the sustainable production method to produce emulsifiers that meet consumers' demands and streamline industrial production processes.

Enzymes commonly employed to catalyze the esterification process include lipases from *Candida antarctica* (Ren & Lamsal, 2017), *Candida rugosa* (Karimian et al., 2023), *Thermomyces lanuginosus* (Udomrati & Gohtani, 2013) and *Rhizopus arrhizus* (Murakami, Morimoto, Imamura, Nagatsu, & Sakakibara, 1994), etc. Among them, the lipase from *Candida antarctica* has been extensively studied due to its relatively stable enzymatic activity in the reaction medium and convenient usage of its immobilized form. The enzyme is a type of esterase that follows the ping-pong catalytic mechanism (Anderson, Larsson, & Kirk, 1998): the catalytic triad Ser-Asp-His captures the acyl donor (typically a fatty acid or its vinyl ester) and forms a first acyl-enzyme intermediate, then it releases a water molecule, an acyl-Ser complex and an Asp-His complex. The latter two complexes subsequently bind the acyl acceptor (sugar or alcohol molecules), forming a second intermediate, and finally releases the sugar-fatty acid ester and free enzyme. As the channel towards the catalytic triad for the acyl acceptor is narrower than the one for acyl donor, there exists regioselectivity for the acyl acceptor. Literature suggests the regioselectivity of a certain enzyme for sugar esters was influenced by the polarity of the reaction medium and the substrate structure, which can affect the collision frequency of the substrate and active sites. For example, Ferrer et al. (1999) observed sucrose monoester was the primary product when the reaction medium polarity increased, while sucrose diester content increased when the polarity decreased, as the latter condition limited the diffusion of the sucrose, allowing longer residence in the active site and favoring diester formation. Pedersen, Wimmer, Emmersen, Degn, and Pedersen (2002) reported when the maltose ester reacted with capric acid and lauric acid, only the 6′- position was acylated, but 6- and 6′- positions were acylated when butyric acid was used, possibly due to the smaller steric hindrance of the second acyl-enzyme intermediate with butyric acid.

The regioselectivity of the reaction significantly affects the functionalities of the synthesized SFAE, particularly for their surface activity and antimicrobial properties. Structural variations, including differences in the fatty acid and glycosyl types, acylation positions and the degree of substitution can influence the packing of the molecules at the hydrophobic-hydrophilic interface, thereby affecting surface activity and interaction with microorganisms. Several studies have examined the functionalities of sugar esters with varying fatty acid chain lengths (Ma, Banwell, Yan, & Lan, 2018; Marathe, Shah, & Singhal, 2020; Zhu et al., 2022), degree of substitution (Marathe et al., 2020), and glycosyl structures (Campana et al., 2019; Park, Sledge, Benninghoff, & Walsh, 2023; Zhao, Zhang, Hao, & Li, 2015). Our analysis indicates that shorter and medium chain length (C10-C14) exhibit relatively good emulsification, foaming and antimicrobial properties. The degree of substitution and glycosyl group affected the aqueous solubilities of the molecules which showed positive relationship with the surface activity and antimicrobial efficacy.

One gap in the literature, as perceived by the authors, is the limited exploration of the structural-functional relationships in the esters where the sugar and fatty acid moieties possess special functions or are under-utilized. The effect of glycosyl group on the functional study has also been insufficiently studied. In this context, we selected three monosaccharides epimers (glucose, mannose and galactose) and two disaccharide isomers (maltose and trehalose) to study the regioselectivity and the resulting esters' functionalities. The mannose was reported having superior aqueous solubility and being inhibitory to some *E.coli* strains that cause urinary tract infections (Ala-Jaakkola, Laitila, Ouwehand, & Lehtoranta, 2022). Galactose, which widely exists in dairy wastes, macroalgae and plants and is a low - sweetness sugar, has not been widely utilized as a food additive in the food industry (Chen et al., 2021). Trehalose is used widely in the food industry due to its acid and heat stability. It shows a retarding effect on starch retrogradation and suppresses the bitter taste in food (Marathe et al., 2020). The glucose and maltose moiety were studied extensively, thus they served as comparative molecules. The fatty acid component was limited to lauric acid, as it has demonstrated superior antimicrobial properties compared other fatty acids with varying chain lengths (Perona et al., 2024).

The objectives of the study were to 1) investigate the acylation positions of the esters derived from five different saccharides under the catalysis of lipase from *Candida antarctica* and 2) evaluate functionalities of these synthesized esters as emulsifiers and antimicrobial agents against representative Gram-positive and Gram-negative bacteria that may cause food spoilage, and to compare the properties with commercial sucrose esters of equivalent hydrophile-lipophile balance (HLB) values. The hypothesis was that: 1) the primary acylation positions would be the 6- or the 6′- positions of the sugar structures and 2) the glycosyl groups of the synthesized esters would have a significant impact on the synthesis yield, emulsion-stabilizing effect, foamability, foam stability and antibacterial properties. To accomplish these objectives, the reactions were conducted in mixed solvent system. The synthesized esters were purified and structurally confirmed through HPLC, FTIR, and NMR techniques. Functionalities were assessed using established methods.

## 2 Materials and methods

### 2.1 Reagents

Galactose (>98 %), mannose (>99 %), maltose (>95 %), trehalose (>99 %), vinyl laurate (>99 %), tert-butanol (>99 %), DMSO (>99.8 %), hexane (97 %), methanol (HPLC grade) and acetonitrile (HPLC grade) were purchased from Macklin (Shanghai, China). Glucose (analytical grade) and potassium bromide (analytical grade) were purchased from Sinopharm Chemical Reagent Co., Ltd. (Shanghai, China). Dichloromethane (>99 %) was purchased from Jinling Group (Shandong, China). Lipase from *Candida antarctica* was purchased from Sigma-aldrich (St. Louis, USA). Sucrose esters S-1670 (HLB 16, stearic acid as the hydrophobic moiety, purity 100 %), and L-195 (HLB 1, lauric acid as the hydrophobic moiety, purity 100 %) were purchased from Mitsubishi Group (Tokyo, Japan). Sucrose esters SE-16 (HLB 16, purity 99 %) and SE-1 (HLB 1, purity 99 %) were purchased from Liuzhou Aigefu Food Technology Co. (Guangxi, China). Sucrose esters with the HLB values of 9.94, 9.8 and 13 were mixed by the above materials based on their original HLB values and mass portion (Griffin, 1954). Canola oil was obtained from a local supermarket. Silicon powder and molecular sieves were purchased from Qingdao Haiyang Chemical CO., Ltd. (Shandong, China) and Shanghai Aladdin Biochemical Technology Co., Ltd. (Shanghai, China).

### 2.2 Synthesis of mono- and disaccharide fatty acid esters and estimation of their HLB values

The synthesis method was adapted from a previous study with modifications (Ren & Lamsal, 2017). The lipase-catalyzed synthesis of mono- and disaccharide fatty acid esters were conducted in 50 mL Erlenmeyer flasks with screw caps. The molar ratios for acyl acceptor and acyl donor for the enzymatic reactions were 1 mmol: 1 mmol for monosaccharide fatty acid esters and 0.4 mmol: 1 mmol for disaccharide fatty acid esters synthesis, respectively. The monosaccharides (acyl acceptors) used were glucose, galactose, mannose. The disaccharides were maltose and trehalose. The acyl donor was vinyl laurate. The sugars were added in 2 mL DMSO and swirled until they were mostly dissolved, followed by 8 mL tert-butanol addition. Then 1 g of molecular sie*v*es and 0.1 g of lipase from *Candida antarctica* were added. The flask contents were gently mixed by swirling and was placed in a water bath at temperature of 50 °C, with the shaking speed of 120 rmp for 36 h.

The HLB *v*alues for the synthesized esters were estimated by calculation based on the eq. (1) (Szymanowski, Hirons, & Christopher, 1984), where M_H_ stands for the effective molecular mass of the hydrophilic groups in the molecule, and M represents molecular mass of the ester's molecule. The HLB value for monosaccharide ester (including 6-*O*-laurylglucose, 6-*O*-laurylgalactose and 6-*O*-lauryl mannose) was 9.9, and the HLB for disaccharide ester (including 6′-*O*-lauryl maltose, 6-*O*-lauryl trehalose or 6'-*O*-lauryl trehalose) was 13.05.

(1) $$\mathrm{HLB}=20\times \;M_{H}/M$$

### 2.3 Purification of fatty acid esters

The purification of all the esters were conducted by solvent extraction method (Ren & Lamsal, 2017). Following the 36-h reaction, the mixture was filtered to obtain the liquid portion and then was placed in a rotary evaporator at 50 °C to remove the tert-butanol. Subsequently, distilled water (1:6 (v/v) for monosaccharide ester and 1:2 (v/v) for disaccharide ester) was added into the mixture to dissolve any unreacted sugar. The resulting cloudy solution was centrifuged and filtered to remove water-soluble substances (DMSO and unreacted sugar). The remaining solid was washed three times with methanol (for glucose ester) or hexane (for other esters) to remove organic byproduct (lauric acid). The washed solid was dried at 50 °C in an oven until a constant weight was achieved.

### 2.4 Identification of fatty acid esters

#### 2.4.1 Identification of sugar-fatty acid esters by fourier transform infrared (FT-IR)

Two milligrams of each type of the dried solid obtained from 2.3 section was blended with 0.2 g potassium bromide and pressed into a semi-transparent pellet (diameter of 0.72 cm) and placed on the ATR crystal of a FT-IR spectrophotometer (IRTracer-100, Shimadzu). The transmittance mode was used for the analysis. The samples were scanned 32 times at a resolution of 4 cm^−1^ with the wavenumber range from 4000 to 400 cm^−1^.

#### 2.4.2 Identification of sugar-fatty acid esters by liquid chromatography mass spectrometry (LCMS)

The Agilent 1260–6125 liquid chromatography – triple quadrupole mass spectrometer was used for measuring the molecular weight of the sugar fatty acid esters. A Zorbax SB-C18 column (4.6 × 250 mm, 5 μm) and an electrospray ionization detector from 2 to 2000 *m*/*z* were used. The samples were scanned in negative mode. An isocratic mobile phase (methanol: water (4:1, v/v) with 0.1 % v/v formic acid) was kept for 30 min and the flow rate was 1 mL/mL. The column temperature was set at 35 °C.

#### 2.4.3 Identification of sugar-fatty acid esters by nuclear magnetic resonance spectroscopy (NMR) and determination of the esters' purity

Heteronuclear multiple bond correlation (HMBC) spectroscopy for 1H and 13C of nuclear magnetic resonance (NMR) (Bruker Avance Neo 400 mHz, Billerica,MA and Karlsruhe, Germany) was conducted to confirm the formation of ester bonds and chemical shifts of purified sugar-fatty acid esters. The specimens were dissolved in deuterated DMSO at a concentration of approximately 10 mg/mL. The data were analyzed with MestReNova software.

The major impurities in the sugar esters were unreacted vinyl laurate and free lauric acid (by-product). The purity was determined by eq. (2).

(2) $$\;\mathrm{Purity of ester}\;\left(\%\right)=\frac{A_{2.29}}{A_{2.4}+A_{2.29}+A_{2.18}}\times 100\%$$

where A_2.29_, A_2.4_, A_2.18_ are the area of the triplet peaks at around 2.29 ppm, 2.40 ppm, 2.18 ppm, respectively. These are the chemical shifts of the α‑hydrogen of the α‑carbon linked to the carbonyl of esters, vinyl laurate and lauric acid.

The following data present the chemical shifts, splitting pattern, J-coupling, positions of hydrogen and carbon atoms and molecular weights for synthesized sugar-fatty acid esters.

6-*O*-laurylglucose.

^1^H NMR (400 MHz, DMSO‑*d*_6_) *δ* 4.89 (t, *J* = 4.2 Hz, 1H, H-1), 4.26 (dd, *J* = 11.7, 2.0 Hz, 1H, H-6a), 3.98 (dd, *J* = 11.7, 6.2 Hz, 1H, H-6b), 3.76 (ddd, *J* = 10.0, 6.2, 2.0 Hz, 1H, H-5), 3.42 (td, *J* = 9.2, 4.7 Hz, 1H, H-3), 3.11 (ddd, *J* = 9.8, 6.7, 3.5 Hz, 1H, H-2), 3.02 (ddd, *J* = 10.0, 8.7, 5.7 Hz, 1H, H-4), 2.27 (td, *J* = 7.3, 1.2 Hz, 2H, -CH_2_-CO-), 1.50 (m, 2H, -CH_2_-CH_2_-CO-), 1.23 (s, 16H, chain), 0.85 (m, 3H, -CH_3_). ^13^C NMR (101 MHz, DMSO‑*d*_6_) *δ* 172.96 (C=O), 92.31 (C-1), 72.87 (C-3), 72.20 (C-2), 70.56 (C-4), 69.15 (C-5), 63.89 (C-6), 33.68 (-CH_2_-CO-), 33.46, 31.31, 29.02, 28.93, 28.75, 28.56, 28.46, 24.51, 22.12 (-CH_2_- lauroyl backbone), 13.97 (-CH_3_). MS (ESI): *m*/*z* 361.2[M-H]^−^, 397.1[M + Cl]^−^.

6-*O*-laurylgalactose.

^1^H NMR (600 MHz, DMSO‑*d*_6_) *δ* 4.52 (s, 1H, H-1), 4.06 (d, *J* = 5.6 Hz, 1H, H-6a), 4.04 (s, 1H, H-5), 3.98 (t, *J* = 6.7 Hz, 1H, H-6b), 3.66 (d, *J* = 11.7 Hz, 1H, H-4), 3.55 (m, 1H, H-3), 3.50 (dd, *J* = 10.0, 3.6 Hz, 1H, H-2), 2.26 (t, *J* = 7.5 Hz, 2H, -CH_2_-CO-), 1.49 (dq, *J* = 15.1, 7.4, 7.0 Hz, 2H, -CH_2_-CH_2_-CO-), 1.23 (s, 16H, chain), 0.85 (t, *J* = 6.9 Hz, 3H, -CH_3_). ^13^C NMR (101 MHz, DMSO‑*d*_6_) *δ* 172.92 (C=O), 92.66 (C-1), 69.30 (C-5), 69.00 (C-4), 68.49 (C-3), 67.64 (C-2), 64.07(C-6), 33.43 (-CH_2_-CO-), 31.34, 29.04, 28.95, 28.79, 28.75, 28.59, 28.50 (-CH_2_- lauroyl backbone), 24.53 (-CH_2_-CH_2_-CO-), 22.14 (-CH_2_-lauroyl backbone), 13.98 (-CH_3_). MS (ESI): *m*/*z* 361.2[M-H]^−^, 397.1[M + Cl]^−^.

6-*O*-lauryl mannose.

^1^H NMR (400 MHz, DMSO‑*d*_6_) *δ* 4.88 (S, 1H, H-1), 4.28 (dd, *J* = 11.6, 1.9 Hz, 1H, H-6a), 3.98 (dd, *J* = 11.5, 6.9 Hz, 1H, H-6b), 3.70 (ddd, *J* = 9.2, 7.0, 1.9 Hz, 1H, H-5), 3.57–3.52 (m, 2H, H-2, H-3), 3.50 (dt, *J* = 12.3, 3.4 Hz, 1H, H-4), 2.26 (t, *J* = 7.3 Hz, 2H, -CH_2_-CO-), 1.50 (p, *J* = 6.9 Hz, 2H, -CH_2_-CH_2_-CO-), 1.24 (s, 16H, chain), 0.83 (t, *J* = 6.7 Hz, 3H, -CH_3_). ^13^C NMR (101 MHz, DMSO‑*d*_6_) *δ* 173.03 (C=O), 94.07 (C-1), 71.31 (C-5), 70.41 (C-2), 70.36 (C-3), 67.15 (C-4), 64.26 (C-6), 33.47 (-CH_2_-CO-), 31.33, 29.04, 28.94, 28.77, 28.75, 28.51, 28.47 (-CH_2_- lauroyl backbone), 24.48 (-CH_2_-CH_2_-CO-), 22.13 (-CH_2_-lauroyl backbone), 13.98 (-CH_3_). MS (ESI): *m*/*z* 361.2[M-H]^−^, 397.1[M + Cl]^−^.

6′-*O*-lauryl maltose.

^1^H NMR (400 MHz, DMSO‑*d*_6_) *δ* 4.89 (d, *J* = 3.7 Hz, 1H, H-1′), 4.26 (dd, *J* = 11.7, 2.0 Hz, 1H, H-1), 3.98 (dd, *J* = 11.7, 6.2 Hz, 2H, H-6’a, H-6’b), 3.76 (ddd, *J* = 8.6, 6.4, 2.0 Hz, 2H, H-5′, H-6a), 3.50 (s, 1H, H-6b), 3.40 (d, *J* = 20.5 Hz, 3H, H-3, H-3′, H-4), 3.12 (dd, *J* = 9.5, 3.7 Hz, 2H, H-2′, H-5), 3.03 (t, *J* = 9.4 Hz, 2H, H-4′, H-2), 2.26 (t, *J* = 7.3 Hz, 2H, -CH_2_-CO-), 1.48 (h, *J* = 6.6 Hz, 2H, -CH_2_-CH_2_-CO-), 1.23 (s, 16H, -CH_2_- chain), 0.85 (t, *J* = 6.6 Hz, 3H, -CH_3_). ^13^C NMR (101 MHz, DMSO‑*d*_6_) *δ* 173.02 (C=O), 96.94 (C-1′), 92.34 (C-1), 76.44 (C-4), 74.72 (C-3′), 73.55 (C-5), 72.90 (C-2), 72.23 (C-3), 70.59 (C-5′), 70.18 (C-2′), 69.84 (C-4′), 69.18 (C-6), 63.91 (C-6′), 33.73 (-CH_2_-CO-), 31.36, 29.06, 28.97, 28.80, 28.77, 28.60, 28.50, (-CH_2_- lauroyl backbone), 24.55 (-CH_2_-CH_2_-CO-), 22.15 (-CH_2_-lauroyl backbone), 14.00 (-CH_3_). MS(ESI):*m*/*z* 523.3[M-H]^−^.

6-*O*-lauryl trehalose or 6'-*O*-lauryl trehalose.

^1^H NMR (400 MHz, DMSO‑*d*_6_) *δ* 4.85 (dd, *J* = 11.9, 3.4 Hz, 2H, H-1, H-1′), 4.22 (d, *J* = 11.6 Hz, 1H, H-6a), 4.02 (dd, *J* = 11.8, 5.5 Hz, 1H, H-6b), 3.91–3.87 (dd, *J* = 10.5, 5.3 Hz, 3H, H-5, H-5′), 3.68–3.41 (m, 4H, H-6’a, H-6’b, H-3, H-3′), 3.25 (ddd, *J* = 9.5, 5.6, 3.7 Hz, 2H, H-2, H-2′), 3.12 (t, *J* = 9.1 Hz, 2H, H-4, H-4′), 2.25 (tt, *J* = 41.5, 7.4 Hz, 2H, -CH_2_-CO-), 1.48 (dq, *J* = 13.3, 6.7 Hz, 2H, -CH_2_-CH_2_-CO-), 1.22 (s, 16H, chain), 0.83 (t, *J* = 6.6 Hz, 3H, -CH_3_). ^13^C NMR(101 MHz,DMSO‑*d*_6_) *δ*172.94 (C=O), 97.92 (C-1′), 93.42 (C-1), 72.95 (C -2′), 72.90 (C-2), 72.69 (C-5′), 71.68 (C-3, C-3′),70.25 (C-4, C-4′), 69.76 (C-5), 63.21 (C-6), 60.93 (C-6′), 33.86 (-CH_2_-CO-), 33.65 (-CH_2_-CH_2_-CH_3_), 33.16, 31.47, 29.22, 29.18, 29.15, 29.08, 24.66, (-CH_2_-,lauroyl backbone), 22.26 (-CH_2_-lauroyl backbone),14.05 (-CH_3_).

MS(ESI):m/z 440.1[M-CH_2_CH_2_CH_2_CH_2_CH_2_CH_3_]^−^, m/z 523.3[M-1]^−^.

### 2.5 Quantification of sugar-fatty acid esters synthesis

The quantification of sugar-fatty acid esters was performed by an in-house de*v*eloped High-Performance-Liquid-Chromatography (HPLC) method. The purified esters were used as standards. At 3 h, 12 h, 24 h and 36 h, aliquots of reactant mixtures were withdrawn, diluted and injected into an HPLC system (Shimadzu Europa, LC-20 A) equipped with an ELSD detector (Alltech, 2000 ES) and a C18 column (Water, Symmetry). The tube temperature and gas flow were set at 90 °C and 1.6 L/min respectively. The mobile phase A (water) and B (acetonitrile) (50/50, v/v) were used as initial condition, then a gradient from this eluent to the ratio of 10/90 (v/v) was continued for 5 mins and maintained for 5 min. After that, the mobile phase was changed to pure acetonitrile in 1 min and lasted for 5 min, then the gradient turned back to A/B 50/50 and equilibrated for 30 min. The column temperature was 35 °C and the flow rate was 1 min/mL. The esters yield was calculated following the eq. (3):

(3) $$\mathrm{Yield ester}\;\left(\%\right)=\frac{\text{synthesized ester amount}\;\left(\mathrm{mol}\right)}{\text{acyl acceptor amount placed in the medium}\;\left(\mathrm{mol}\right)}\times 100\%$$

### 2.6 Functionalities study

#### 2.6.1 Emulsion preparation and particle size analysis

Each in-house synthesized and commercial sugar-fatty acid ester was placed in the canola oil and sonicated until dispersed, then distilled water was added. The mixture was homogenized at 12000 rpm for 2 min. The concentrations of the emulsifiers and oil were 0.5 % (w/w) and 4 % (w/w), respectively. The resulting emulsions were stored under 25 °C and 4 °C for 7 days. At 0 h, 24 h, 3 d and 7 d, proper amount of samples was withdrawn to conduct particle size analysis and zeta-potential test.

The particle size analysis was measured by a particle size analyzer (Beckman, LS 13320). Samples from the aforementioned emulsions were placed in the sample chamber until the degree of obscuration reached 5 %. The D[10], D[50], D[90], D[4,3] and size distribution were recorded and analyzed.

#### 2.6.2 Foamability and foam stability

The foamability and foam stability tests were conducted following published studies with modifications (Zhu et al., 2022). Sugar-fatty acid esters' aqueous solutions were prepared with the concentration of 0.5 % (w/w). Thirty milliliter solution was placed in a 100 mL centrifuge tube and the height of the solution was recorded as H_0_. The solution was homogenized (IKA, T18 digital) for 30 s at 5000 rpm and continued for 30 s at 12000 rpm, the height of the generated foam was recorded as H_1_. At 20 min, 40 min, 80 min and 120 min, the heights of the foam were recorded as H_n_ (*n* = 20, 40, 80, 120). The foamability and foam stability were calculated based on the eq. (4), (5):

(4) $$\text{Foamability}\;\left(\%\right)=\left(H_{1}/H_{0}\right)\times 100\%$$

(5) $$\text{Foam stability}\;\left(\%\right)=\left(H_{n}/H_{0}\right)\times 100\%$$

#### 2.6.3 Zeta-potential analysis

The zeta potential *v*alues of the emulsions were measured by a dynamic light scattering instrument (Malvern, Zeta sizerNano ZS). The emulsion samples were diluted in phosphate buffer solution (0.005 M sodium dihydrogen phosphate and 0.005 M disodium hydrogen phosphate in 1 L distilled water, pH 7.0) in a ratio of 1:500 (v/v). One milliliter of diluted emulsion was placed in a sample cuvette, and three measurements were taken for each sample.

#### 2.6.4 Antimicrobial properties

The five in-house synthesized sugar esters were tested for antimicrobial efficacy against two representative Gram-positive and Gram-negative species *Bacillus subtilis* 5009 and *Escherichia coli* 12024. Bacterial stock cultures were stored at −80 °C and activated by inoculating on Luria-Bertani (LB) plate. The bacteria were then subcultured in LB broth at 37 °C for 24 h. After plate counting, the bacteria suspensions were adjusted to obtain the cell count around 2 × 10^7^ CFU/mL.

The sugar-fatty acid esters solutions were prepared as follows. The glucose ester, maltose ester and trehalose ester, which were not completely water soluble, were initially dissolved in DMSO and diluted with LB broth (DMSO 1 %, v/v). The galactose ester and mannose ester were directly dissolved in LB broth. The stock solutions for all the sugar esters were 8 mg/mL. Proper amount of the stock solutions was mixed with bacterial broth to reach the esters' final concentration to 4 mg/mL, 2 mg/mL, 1 mg/mL, 0.5 mg/mL, 0.25 mg/mL and 0.125 mg/mL. Two hundred microliters of the mixed bacterial solutions were placed in the wells of a microplate which was placed in a microplate reader (Molecular De*v*ices, SpectraMax i3X). At 0 h, 3 h, 9 h, 14 h and 24 h, absorbance values at 600 nm were recorded. Control and experimental groups were designated as follows: Control 1 (C1) – sugar ester solutions, Control 2 (C2)– bacterial solution without sugar ester, Control 3 (C3) – LB broth, Experimental group (E) – bacterial solution with sugar ester. The minimum inhibitory concentration (MIC) was defined as the lowest concentration that can inhibit bacterial growth during 24 h, which was shown as no increase in the optical density (OD) during 24 h. The inhibition percentage was defined as the eq. (6) (Ren, Patra, Lamsal, & Mendonca, 2021):

(6) $$\;\mathrm{Inhibition effect}\;\left(\%\right)=\frac{\mathrm{OD}\left(24\right)-\mathrm{OD}\left(\mathrm{sugar ester}\;24\right)}{\mathrm{OD}\left(24\right)}\times 100\%$$

where OD (24) is the difference in OD values between Control 2 and 3 (C2 – C3) at 24 h, OD (sugar ester 24) is the differences in OD values between experimental group and Control 1 (*E*-C1). For glucose ester, maltose ester and trehalose ester, all control and experimental groups contained 1 % DMSO (v/v); the other two esters did not.

### 2.7 Statistical analysis

Three samples were prepared for sugar-fatty acid esters synthesis, HPLC quantification and each functionality study. For antimicrobial test, there were also three samples prepared for each treatment group. One-way ANOVA test was used to analyze the esters' synthesis. Three-way ANOVA analysis was conducted for functionality studies. Data were analyzed by SPSS 26.0 software (IBM).

## 3 Results and discussion

### 3.1 Sugar-fatty acid esters' identification and 1H and 13C assignment for products

**Fig. 1:**
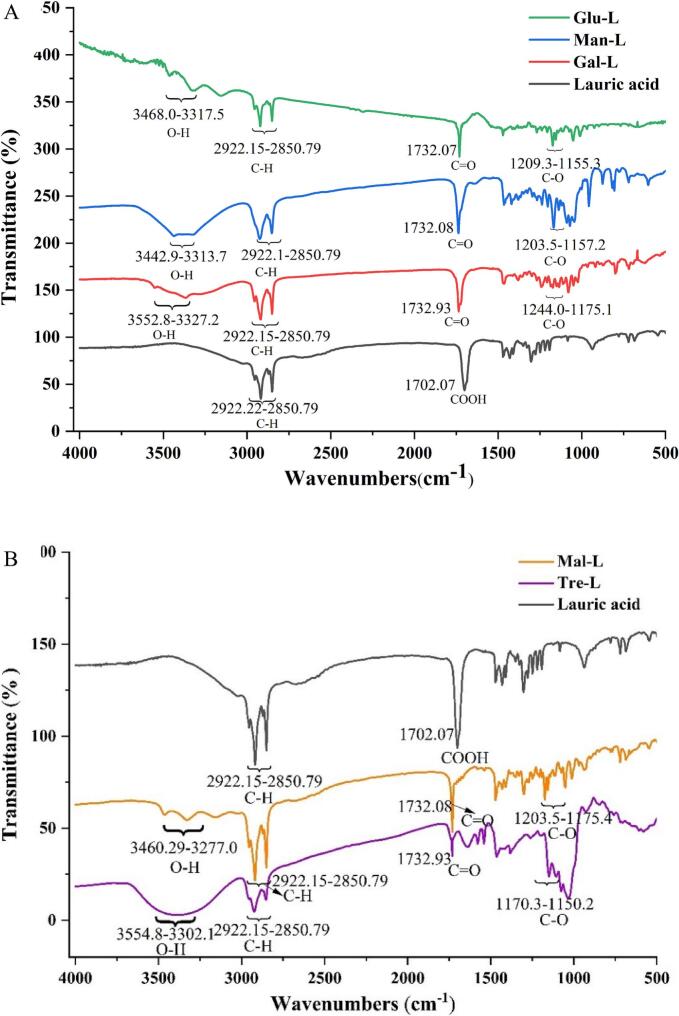
Fourier infrared spectra of monosaccharide esters (A) and disaccharide esters (B). Glu-L: *6-O-lauryl glucose*; Gal-L: *6-O-lauryl galactose*; Man-L:*6-O-lauryl mannose*; Mal-L: *6′-O-lauryl maltose*; Tre-L: *6-O-lauryl trehalose* and *6′-O-lauryl trehalose.*

The identification of sugar-fatty acid esters was performed by Fourier infrared spectra (Fig. 1) and nuclear magnetic resonance techniques. In FTIR, the broad region around 3570–3270 cm^−1^ corresponded to the stretching vibrations of alcohol hydroxyl groups. The splitting pattern observed around 2922–2850 cm^−^1 was attributed to the stretching vibrations of methyl and methylene groups. The sharp adsorption peaks around 1732 cm^−1^ indicated the carbonyl ester adsorption frequency. The adsorption peaks in the region of 1150–1250 cm^−1^ were indicative of the stretching vibrations of C—O bond of ester and tertiary alcohol of the sugar (Gao, 2005).

**Fig. 2:**
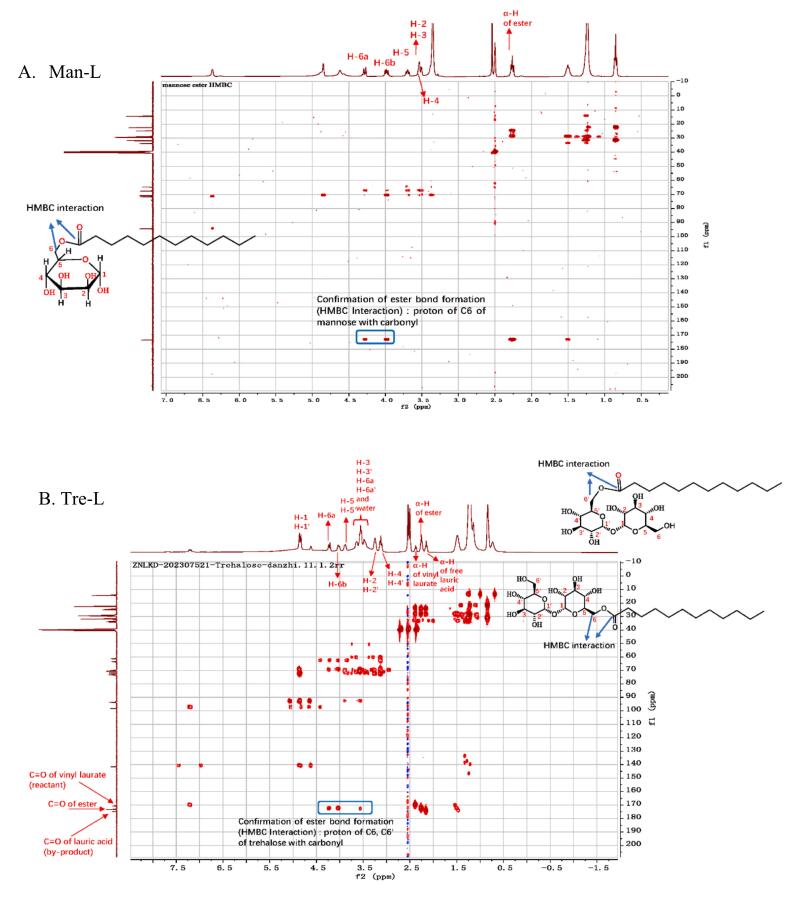
Heteronuclear multiple bond (HMBC) of purified *6-O-lauryl mannose* (A. Man-L) and *6-O-lauryl trehalose and 6′-O-lauryl trehalose* (B, Tre-L).

The sugar esters' structures were further confirmed by the magnetic resonance technique. The heteronuclear multiple bond correlation (HMBC) was used to verify the ester bond formation between the hydroxyl group(s) of the sugar molecules and the carbonyl atom of the vinyl laurate. Once the ester bond was formed, an interaction on the two-dimensional spectra can be observed. Ester bonds were formed for every sugar used in this study. It was found the bond was exclusively formed at the 6-OH for glucose, galactose and mannose. For maltose esters, the 6’-OH was acylated. For trehalose, it is deduced that the ester bond was formed either with 6-OH or 6'-OH. As HMBC interactions were found for these two positions and LCMS data indicated only monoester existed. Additionally, only one type of α‑hydrogen of ester was found. Representative HMBC spectra for one monosaccharide ester (6-*O*-mannose monoester) and one disaccharide ester (6-*O*-trehalose monoester/6'-*O*-trehalose monoester) were presented (Fig. 2A and B), others were in the Supplementary Fig. S1. The purities of glucose monolaurate (Glu-L), galactose monolaurate (Gal-L), mannose monolaurate (Man-L), maltose monolaurate (Mal-L) and trehalose (Tre-L) were 88.5 %, 71.1 %, 93.5 %, 31.50 % and 43.4 %, respectively. The impurity substance in Glu-L, Gal-L and Man-L was lauric acid. While in Mal-L and Tre-L, the impurity substances were lauric acid and vinyl laurate. These larger structures can dissolve more hydrophobic vinyl laurate, thus making the separation more difficult.

### 3.2 Sugar-fatty acid esters yield

**Fig. 3:**
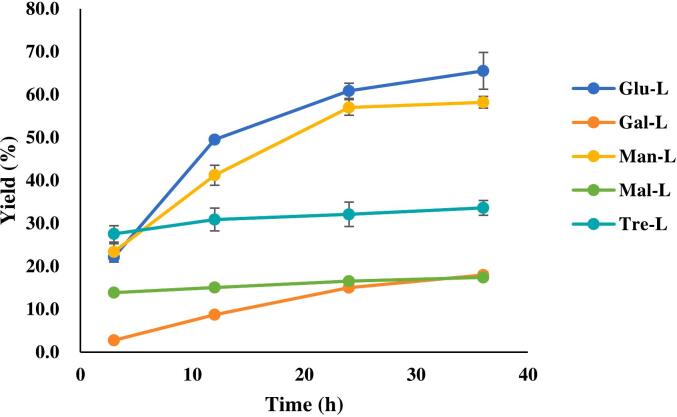
The yield of five types of sugar esters over 36 h. Glu-L: *6-O-lauryl glucose*; Gal-L: *6-O-lauryl galactose*; Man-L*: 6-O-lauryl mannose*; Mal-L:*6′-O-lauryl maltose*; Tre-L:*6-O-lauryl trehalose and 6′-O-lauryl trehalose*

The study of yields and reaction rates is crucial for industrial synthesis. The sugar-fatty acid esters yield (%) at 3 h, 12 h, 24 h and 36 h were depicted in Fig. 3. For each ester, the yield gradually increased, while the rate of synthesis declined over time. The yield of Tre-L, Man-L and Glu-L approached equilibrium during 24–36 h (*P* > 0.05), while the Mal-L and Gal-L were slowly increasing and reached the highest point at 36 h (*P* < 0.05). The final yield varied significantly among the esters, with the Glu-L (65.49 %) and Man-L (58.16 %) being the highest, followed by Tre-L (33.58 %), Gal-L (17.94 %) and Mal-L (17.38 %). According to literature, the substrates solubility in the medium greatly influenced the yield and production rate (Ferrer et al., 1999; Ren & Lamsal, 2017). Based on several studies, the solubility of mannose, glucose and galactose in water were 2.6 g/g, 0.90 g/g and 0.40 g/g (Jäntschi, 2019). Tatsuoka and Yamaguchi (2023) reported that mannose had the most affinity to the nearby water molecules compared to glucose and galactose. Gong, Wang, Zhang, and Qu (2012) reported the maltose and trehalose solubility in water were 0.4548 g/g and 0.4448 g/g, respectively. Although our system was non-aqueous, the solvent system (DMSO and tert-butanol) offered some hydrophilicity, thus the solubility rank can explain the yields ranking to some degree. For example, the galactose had the lowest aqueous solubility and thus the yield was also the lowest. However, the structures of the sugar should also be taken into consideration. For instance, despite mannose having the highest aqueous solubility, the ester's yield was lower than that of glucose. These two sugar molecules differ in the orientation of the hydroxyl group of the C-2, which may affect the access to the enzyme active site thus give rise to the different catalytic efficiency. Trehalose and maltose are isomers which are composed of different monosaccharides. Although they had similar solubilities, the former one achieved almost twice fold yield of the latter one. The exact reasons contributing to these catalytic differences were not elucidated clearly from the authors' literature review, thus some novel technologies, such as molecular docking and molecular simulations, are suggested to study the mechanisms.

### 3.3 Particle analysis of the emulsions

Table 1 presents the D[4,3] values of emulsions stabilized by in-house synthesized sugar esters and commercial esters. The effect of temperature, esters' type and time and their interactions on the particle size were analyzed. It was determined the type, time, temperature*time, type*time had significant impact on the particle size (*P* < 0.05).

**Table 1:** D[4,3] values of different emulsions at two temperatures (μm).

Emulsifier	Fresh emulsion	25 °C			4 °C		
		24 h	3 d	7 d	24 h	3 d	7 d
Glu-L	2.8 ± 0.1^a^	3.1 ± 0.5^a^	2.9 ± 0.03^abc^	2.8 ± 0.4^b^	3.3 ± 0.4^ab^	2.9 ± 0.2^bc^	2.8 ± 0.2^c^
Gal-L	1.9 ± 0.1^ab^	2.2 ± 0.2^a^	1.9 ± 0.03^a^	1.8 ± 0.1^a^	1.9 ± 0.1^a^	1.9 ± 0.1^a^	2.1 ± 0.4^abc^
Man-L	2.6 ± 0.5^ab^	2.9 ± 0.5^a^	2.8 ± 0.3^abc^	2.9 ± 0.4^a^	3.0 ± 0.3^a^	2.6 ± 0.4^abc^	2.7 ± 0.3^bc^
Mal-L	2.6 ± 0.1^ab^	2.9 ± 0.2^a^	2.7 ± 0.4^ab^	3.0 ± 0.1^b^	2.5 ± 0.1^a^	2.1 ± 0.2^ab^	2.0 ± 0.1^ab^
Tre-L	2.7 ± 0.2^ab^	2.8 ± 0.2^a^	2.7 ± 0.3^ab^	2.5 ± 0.2^ab^	2.5 ± 0.1^a^	2.0 ± 0.1^ab^	1.9 ± 0.1^a^
SE1(9.8)	6.1 ± 1.2^d^	5.7 ± 1.1^b^	4.5 ± 1.1^bcd^	4.3 ± 0.4^d^	7.1 ± 0.3^cd^	4.2 ± 0.6^de^	5.2 ± 0.1^d^
SE2(9.8)	5.3 ± 1.0^cd^	5.6 ± 0.3^b^	4.6 ± 0.3^bcd^	4.7 ± 0.2^d^	6.1 ± 0.8^c^	4.1 ± 0.7^de^	4.2 ± 0.5^d^
SE1(13)	7.1 ± 0.2^d^	5.3 ± 0.5^b^	4.2 ± 0.5^bcd^	5.4 ± 0.5^e^	8.1 ± 0.7^d^	3.3 ± 1.0^cd^	5.0 ± 1.3^e^
SE2(13)	5.4 ± 0.3^d^	5.6 ± 0.5^b^	5.0 ± 0.6^cd^	4.7 ± 0.2^d^	5.3 ± 1.5^c^	4.9 ± 0.6^ef^	4.6 ± 0.2^de^
SE1(16)	6.3 ± 2.0^d^	5.9 ± 1.7^b^	5.7 ± 2.8^d^	6.5 ± 1.1^de^	6.4 ± 2.4^cd^	5.3 ± 0.2^f^	6.2 ± 0.5^f^
SE2(16)	4.2 ± 0.2^bc^	4.7 ± 0.5^b^	4.2 ± 0.5^Bcd^	4.6 ± 0.2^c^	6.0 ± 1.0^cd^	4.3 ± 0.4^de^	4.7 ± 0.4^de^

Note: different letters indicate significant differences among sugar esters at the same time point and the same temperature (P < 0.05). Glu-L: *6-O-lauryl glucose*; Gal-L: *6-O-lauryl galactose*; Man-L: *6-O-lauryl mannose*; Mal-L:*6′-O-lauryl maltose*; Tre-L:*6-O-lauryl trehalose* and *6′-O-lauryl trehalose*; SE1: sucrose esters from Mitsubishi Group; SE2: sucrose esters from Aigefu Food Technoogy; the numbers in the bracket indicate the HLB value.

The emulsions stabilized by in-house synthesized sugar esters had smaller particle sizes (1.9–3.0 μm) than those of commercial sucrose (3.3–7.1 μm). This difference was probably attributed to the in-house synthesized esters being monoesters, whereas commercial sucrose esters were a mixture of mono- and diesters. The stronger hydrophobic interaction of the diesters with the oil allowed for more oil to be incorporated into the emulsion particles. Despite the larger particle sizes of emulsions stabilized by commercial sucrose esters, their appearance (Supplementary Fig. S2) suggested greater stability compared to those stabilized by in-house synthesized esters. The commercial sucrose ester-stabilized emulsions remained in a cloudy homogeneous state over 7 days, while some oil separation was observed in the in-house synthesized ester-stabilized emulsions by the 3rd day. It is likely that the diesters in the commercial sucrose esters provided some steric hindrance and viscoelasticity on the oil-water interface, which prevented the coalescence. The in-house synthesized monoesters and the impurity substance lauric acid can both serve as emulsifiers. However, these smaller molecules provided less steric hindrance than the diesters in the commercial sucrose esters. Thus, the coalescence phenomenon was more pronounced. Among the three monosaccharide esters, the Glu-L and Man-L had similar particle sizes (around 2.6–2.9 μm) over 7 days, while Gal-L had smaller ones (1.8–1.0 μm). These data suggest the interfacial areas in the three emulsion systems varied, and the Gal-L system had largest interfacial area. The varying configurations and orientations of the sugar esters, as well as the differing concentrations of lauric acid, may contribute to the observed differences in particle sizes. For the Mal-L and Tre-L, the former one had slightly larger particles (2.0–3.0 μm) compared to the latter one (1.9 μm–2.7 μm). The higher purity of the Tre-L probably give rise to the higher interface area in the emulsion system. Overall, the above data showed that at the same HLB value for the in-house synthesized monoesters, the structure of the glycosyl group only had a minor impact on the particle size.

The interaction of time and temperature had a significant impact on the particle size of the emulsions. At the 3rd day, the sizes were 3.75 μm at 25 °C, which was slightly larger than those at 4 °C (3.25 μm). By the 7th day, the differences in the particle sizes became pronounced, with the values of 4.4 μm at 25 °C and 3.8 μm at 4 °C. The phenomenon was anticipated, as the emulsion droplets had higher collision rate at higher temperatures, leading to increased coalescence. The interaction between type and time also influenced the particle size. For instance, the in-house synthesized ones all experienced a slightly decreasing trend over the 7 days, while the commercial sucrose esters stabilized droplets all decreased on the 3rd day, followed by an increase on the 7th day.

Particle size analysis alone may not fully elucidate the stability of emulsions, as flocculated particles that contribute to the formation of an oil layer may exceed the measurement range of the instrument, resulting in the detection of only smaller particles. Matsumiya, Takahashi, Inoue, and Matsumura (2010) observed a similar phenomenon when studying milk stability with sucrose esters. Jeong, Kim, Echeverria-Jaramillo, and Shin (2021) also reported that emulsion particles stabilized by ethyl lauroyl arginate and Tween 80 decreased over 15 days, with cream layers being observed. Consequently, predicting stability should involve the integration of results from other techniques.

### 3.4 Zeta potential of the emulsion particles

**Fig. 4:**
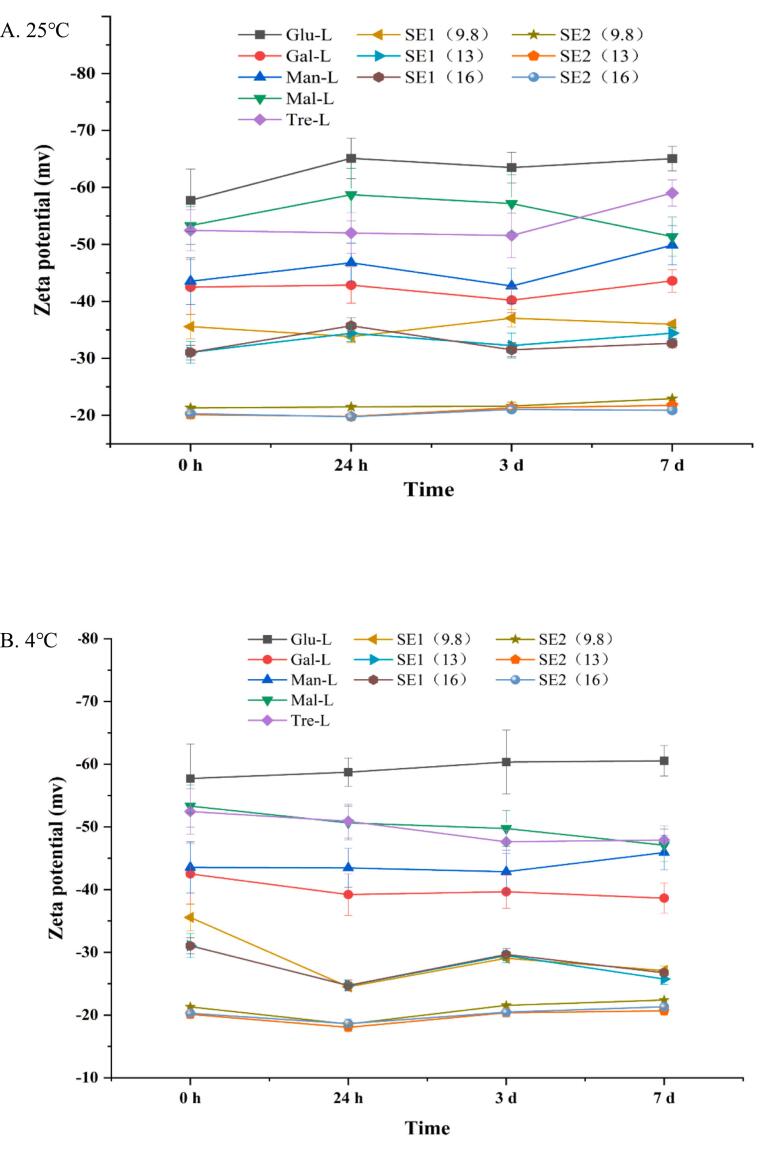
Zeta potential of emulsions particles stabilized by different esters at 25 °C (A) and 4 °C (B). Glu-L: *6-O-lauryl glucose*; Gal-L*: 6-O-lauryl galactose*; Man-L:*6-O-lauryl mannose*; Mal-L: *6′-O-lauryl maltose*; Tre-L: *6-O-lauryl trehalose* and *6′-O-lauryl trehalose*. SE1-sucrose esters purchased from Mitsubishi Group. SE2-sucrose esters purchased from liuzhou aigefu food technology Co. The numbers in the bracket indicate the HLB values.

Zeta potential values represent the effective surface potential of the emulsion particles in the medium, which considers the adsorbed layers (Attama et al., 2023). In our study, the hydroxyl groups of the emulsifiers induced a secondary layer of OH^−^ ions from the aqueous phase, thus negative charges were observed (Fig. 4). The zeta potential can not only measure the charges on the particles, but also can indicate the emulsifiers' surface coverage. Since the oil contains impurities such as free fatty acids, which can act as emulsifiers in oil-in-water emulsions, control samples (without any sugar esters) also exhibited negative charges. Therefore, the comparison between the control samples and experimental samples provides insight into the surface coverage of the sugar esters.

The main effect of temperature, type and time, as well as their interactions on the zeta-potential were analyzed. Notably, all factors except time had significant effects. When comparing the in-house and commercial emulsifiers, the ranking of the absolute zeta potential values was as follows: Glu-L (61.0 mV), Mal-L (52.3 mV), Tre-L (51.2 mV), Man-L (44.8 mV), Gal-L (41.2 mV), SE1 (range 30.0–32.0 mV) and SE2 (range 20–21.4 mV). The absolute zeta potential for the control was 48.9 mV. These values indicate that commercial sucrose esters had greater surface coverage, with the fatty acids of the sucrose esters masking the free fatty acids on the oil droplets, thereby reducing the absolute zeta potential. Similar phenomenon from other studies were also reported (Grgić et al., 2022). Conversely, the in-house synthesized ones exhibited less surface coverage, which can explain that the in-house synthesized emulsifier stabilized emulsions were less stable (Supplementary Fig. S2). The observations of the emulsion particle sizes, zeta potential and the appearance indicated the major stabilizing mechanisms were steric hindrance on the interface.

Among the in-house synthesized emulsifiers, the Man-L and Gal-L exhibited higher surface coverage than others, suggesting these two might be more surface-active than others. The existence of the impurity substance lauric acid also have hydrophobic interaction with the oil droplets, so the surface coverage of Man-L and Gal-L were higher than the Glu-L. There were no significant differences between the Mal-L and Tre-L, indicating the two glycosyl groups may reorient or pack similarly on the water-oil interface. The Glu-L had an even higher zeta-potential than the control emulsion, which was unexpected. Considering the compound is less dispersible than others, it is reasonable to assume the Glu-L was not surface active. It was the compound itself that induced the charges in the emulsion.

The absolute zeta potential values also exhibited significance at different temperatures: 38.6 mV at 25 °C and 35.4 mV at 4 °C. This can be attributed to the increased adsorption density at the droplet interface: as more coalescence or phase separation occurred at higher temperature, the dispersed volume fraction decreased and the number of charges on the interface increased (Bhattacharjee, 2016). The time-type-temperature interaction was also significant, which can be reflected as the curves for different esters at multiple time points under two temperatures (Fig. 4a and b). However, the range for each type did not fluctuate drastically, indicating the interfacial structure did not change significantly over 7 days (Juyang & Wolf, 2021).

### 3.5 Foamability and foam stability

**Fig. 5:**
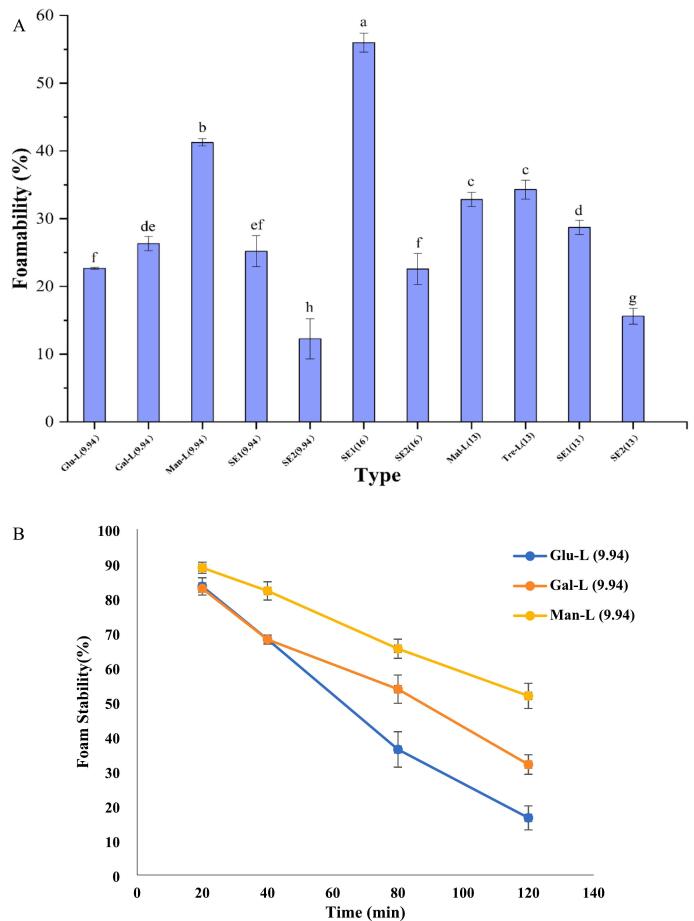

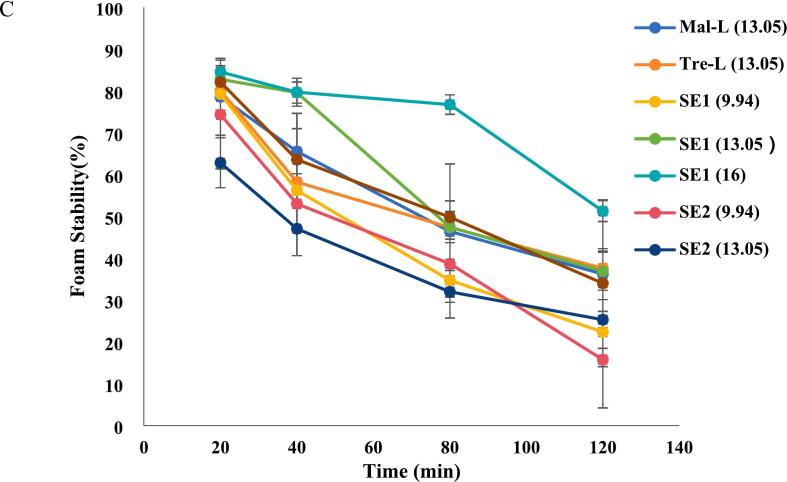
Foamability (A) and foam stability (B, C) for all sugar esters. Glu-L: *6-O-lauryl glucose*; Gal-L*: 6-O-lauryl galactose*; Man-L:*6-O-lauryl mannose*; Mal-L: *6′-O-lauryl maltose*; Tre-L: *6-O-lauryl trehalose* and *6′-O-lauryl trehalose*. SE1-sucrose esters purchased from mitsubishi group. SE2-sucrose esters purchased from Liuzhou Aigefu Food Technology Co. The numbers in the bracket indicate the HLB values.

Foam contributes to the texture and appearance of food, and its formation and stability of foam have a close relationship with surface-active compounds. Therefore, foamability and foam stability were investigated for the esters. The Glu-L, Gal-L, Man-L, Mal-L and Tre-L exhibited the foamability of 22.7 %, 26.3 %, 41.6 %, 32.8 % and 34.3 % respectively (Fig. 5A). Among commercial sucrose esters, SE1 (16) had the highest foamability (56.0 %), others were in the level of 12.3 % - 29 %. Clearly, the SE1(16) and Man-L exhibited the best foamability. All aqueous sugar-ester's solution demonstrated foam instability during the 120 min, which was expected and consistent with a similar study, as low-molecular surface active-compound aqueous solution had low interfacial elasticity (van Kempen, Schols, van der Linden, & Sagis, 2014). Among the in-house synthesized esters, Man-L and Tre-L showed better foam stability, followed by Mal-L and Gal-L (by comparing the curve slopes). Among the commercial sucrose esters, the SE1(16) and SE2(13) had better foam stability than others. The Man-L, Tre-L, SE1(16) and SE2(13) demonstrated the highest stability.

For non-ionic surfactants, foamability and foam stability are influenced by several factors: the migration speed to the surface (measured by the initial surface tension) (Hollenbach et al., 2020; van Kempen et al., 2014), the surfactant exchange rate between adsorption layers and bulk solutions (Mustan et al., 2022; van Kempen et al., 2014) and surface coverage (Petkova et al., 2020). A faster migration speed, or a lower initial surface tension correlates with higher foam stability. van Kempen et al. (2014) reported when the surface tensions were between 20 and 40 mN/m, the sucrose esters achieved the highest foam half time. In our study, the Man-L and Tre-L exhibited relatively better surface activity, as indicated in the zeta-potential section, leading to better foamability and foam stability. The surfactant exchange rate between the interface and the bulk solution should not be too high, which may result in foam destabilization. This requires that the surfactants' concentration exceeds the critical micelle concentration (CMC) level to be able to stabilize the foam. According to documented literature, the CMCs for glucose-, maltose- and trehalose monolaurate were 0.331 mM (Abdelaziz, Ahmed, & Sadek, 2023), 0.232 mM (Ma et al., 2018) and 0.570 mM (Chen et al., 2023). While the CMCs for other esters were not found, the level should be close to the above values. In our study, the concentrations were well above the CMCs, with 13.8 mM for monosaccharide esters and 9.5 mM for disaccharide esters, which meant the destabilization mechanisms for our system were not concentration driven. High surface coverage is more critical for non-ionic surfactants than ionic surfactants for foamability and foam stability, as the former relies on short-range steric interactions for film stabilization, which requires complete surface coverage, whereas the latter benefits from ionic repulsion for additional stability (Petkova et al., 2020). This theory supports our findings: for the in-house synthesized esters, Man-L and Tre-L, with relatively high coverage on the hydrophilic-hydrophobic interface (as shown in zeta-potential section), exhibited the best foam stability among in-house synthesized esters. The commercial SE1 (16) had the best foamability and foam stability, possibly due to the proper mixed ratio of mono- and di-esters, where the monoester had good migration speed to the surface and the diesters provided proper steric hindrance on the interface. Our in-house synthesized Mal-L and Tre-L, although had the same HLB as the SE1 (16), did not show a comparable foamability and foam stability, due to lacking of diesters and lower purities.

Overall, the disaccharide monoesters showed superior foamability and foam stability compared to monosaccharide monoesters. These enhanced properties can be attributed to their faster migration speed to the interface and their greater interfacial coverage, both of which contribute to the mechanical stability of foam films.

### 3.6 Antimicrobial properties of in-house synthesized sugar esters

Sugar esters were known to have antimicrobial efficacy due to the amphiphilic property. At a certain concentration, they formed mixed micelle with the phospholipids of the bacterial cell membrane (Ren et al., 2021), causing surface damage and leakage of cytoplasmic content (Zhao et al., 2015), ultimately leading to cell death. The inhibition percentages of the synthesized five types of esters were measured against *E. coli* 12024 and *Bacillus subtilis* 5009 (Table 2a, Table 2b). The Glu-L showed unsatisfactory antimicrobial effects for both bacteria, likely due to its low aqueous solubility and surface activity, which hinder its attachment to the bacterial surface. The highest inhibition effect was observed at 2 mg/mL for *Bacillus subtilis* (89.63 %), and the inhibition percentages for *E. coli* were generally low (below 31 %). Glucose esters have been shown not very effective against a variety of bacteria. For example, glucose palmitate and glucose laurate monoester did not achieve an MIC to *L.monocytogenes*, *S. enterica* and *E.coli* O157:H7 when the concentrations were up to 4.15–22.1 mg/mL (Ren, 2018). Campana et al. (2019) reported glucose laurate monoester for *E.coli* O157:H7 was not found. Only short-chain glucose ester had been found to have some effects: the glucose monocaprate MICs were 2.5 mM and 10 mM for *B. subtilis* and *E.coli*, respectively (Zhao et al., 2015), probably due to their relatively higher solubility. The Gal-L exhibited good antimicrobial inhibition effect for both bacteria in our study, with the MIC for *E. coli* at 4 mg/mL. Although the MIC for *B. subtilis* was not determined, the concentrations studied (0.125 mg/mL – 4 mg/mL) had the inhibition rate of 84.3 %–99.3 %. Galactose esters have been reported as quite effective antimicrobials for some important bacteria. For example, Jumina, Mutmainah, Purwono, Kurniawan, and Syah (2019) reported the galactosyl monolaurate had an inhibition zone of 4.3 mm on *B. subtilis* and 0 mm on *E.coli*, demonstrating the compound was less effective for *E.coli*, which differs from our findings. Watanabe, Katayama, Matsubara, Honda, and Kuwahara (2000) found the galactose laurate was the most effective sugar ester among 23 types of esters against the dental caries-causing organism *Streptococcus mutans,* with the MIC of only 50 μg/mL, others were at least 100 μg/mL. In our study, Man-L was not as effective as Gal-L at low concentrations (0.125 mg/mL – 1 mg/mL) for both bacteria, and the MICs were found at 4 mg/mL and 2 mg/mL for *E. coli* and *B. subtilis*, respectively. It is possible that the impurity substance, lauric acid, affected the MIC, as it also possesses antimicrobial property (Casillas-Vargas et al., 2021). The purified Man-L had a lower lauric acid content Gal-L. Therefore, at a low level, lauric acid may overpower the antimicrobial effect of the monosaccharide esters, resulting in Gal-L showing better performance. When the concentrations increased, the effect of the monosaccharide esters became dominant. The *B. subtilis* was more susceptible to the Man-L than *E.coli,* probably due to the different compositions of their cell walls. *B. subtilis,* a Gram-positive bacterium, has a cell wall that primarily consists of layers of peptidoglycan containing 1 %–4 % lipid. While *E.coli*, a Gram-negative bacterium, has three-layer cell walls composed of lipoprotein, lipopolysaccharide and peptidoglycan that contain 11 %–12 % lipid (Zhao et al., 2015). Thus, *E.coli* requires more surfactant to form mixed micelles with the phospholipid bilayers, causing surface damage that leads to cellular lysis. The Man-L has been also known as an effective antimicrobial. Campana et al. (2019) found the MIC of mannose laurate against *E.coli* O157:H7 was only 256 μg/mL. In another study, mannose monoesters with the chain lengths of C12-C14 all showed antimicrobial effect against *S. aureus* (ATCC25923 and ATCC33592 MRSA), with the MIC ranging from 16 to 128 μg/mL and C14 being the most effective (MIC 16–32 μg/mL). However, the series of compounds were not as effective against *E.coli* (ATCC25922), the MIC was only found for mannose myristate (128 μg/mL) (AlFindee et al., 2018). Watanabe et al. (2000) reported 6-*O*-laurylmannose was the third effective compounds against *Streptococcus mutans*, with the MIC of 200 μg/mL. It was less effective than galactose laurate (50 μg/mL) and fructose laurate (100 μg/mL). The MICs of glucose and sucrose laurate were not found.

**Table 2a:** Inhibition percentages (%) of in-house synthesized esters against *E.coli* 12024.

	Concentrations (mg/mL)					
Sugar-fatty acid esters	0.125	0.25	0.5	1	2	4
Glu-L	25.9 ± 4.8	10.0 ± 2.2	22.3 ± 9.1	30.3 ± 8.1	21.5 ± 0.7	29.1 ± 1.6
Gal-L	98.0 ± 0.1	93.4 ± 0.5	97.5 ± 1.2	95.5 ± 9.9	96.4 ± 0.7	100.0
Man-L	35.6 ± 0.2	45.8 ± 1.9	57.8 ± 1.4	71.7 ± 8.8	96.8 ± 2.9	100.0
Mal-L	96.6 ± 1.1	90.1 ± 1.0	88.7 ± 3.2	100.0	100.0	100.0
Tre-L	96.2 ± 1.5	87.5 ± 0.7	83.5 ± 4.0	100.0	100.0	100.0
SE	6.2 ± 9.9	31.7 ± 19.2	53.2 ± 2.1	54.6 ± 6.8	69.8 ± 1.4	53.8 ± 4.4

Note: Glu-L: *6-O-lauryl glucose*; Gal-L: *6-O-lauryl galactose*; Man-L: *6-O-lauryl mannose*; Mal-L:*6′-O-lauryl maltose*; Tre-L:*6-O-lauryl trehalose and 6′-O-lauryl trehalose*; SE-sucrose ester (HLB 16) from Mitsubishi Group.

**Table 2b:** Inhibition percentages (%) of in-house synthesized esters against *Bacillus subtilis* 5009.

	Concentrations (mg/mL)					
Sugar-fatty acid esters	0.125	0.25	0.5	1	2	4
Glu-L	10.4 ± 3.7	28.5 ± 1.3	46.5 ± 1.5	53.0 ± 2.6	89.6 ± 2.5	88.7 ± 4.6
Gal-L	95.7 ± 0.7	84.3 ± 3.5	99.3 ± 0.2	92.6 ± 2.1	98.4 ± 4.8	87.1 ± 1.5
Man-L	63.3 ± 0.9	88.2 ± 0.2	90.3 ± 0.3	92.0 ± 6.5	100.0	100.0
Mal-L	95.6 ± 1.9	95.2 ± 1.0	84.8 ± 4.2	98.00 ± 1.6	100.0	100.0
Tre-L	97.6 ± 1.2	91.9 ± 6.8	100.0	100.0	100.0	100.0
SE	5.5 ± 4.8	23.6 ± 12.4	29.8 ± 11.1	92.0 ± 3.3	78.9 ± 0.7	85.7 ± 2.3

Note: Glu-L: *6-O-lauryl glucose*; Gal-L: *6-O-lauryl galactose*; Man-L: *6-O-lauryl mannose*; Mal-L:*6′-O-lauryl maltose*; Tre-L:*6-O-lauryl trehalose and 6′-O-lauryl trehalose*; SE-sucrose ester (HLB 16) from Mitsubishi Group.

Maltose ester and trehalose ester demonstrated superior antibacterial efficacy against both bacteria compared to monosaccharide esters. The inhibition percentages were consistently above 83 % at all concentrations, with the Mal-L' s MIC values being 1 mg/mL and 2 mg/mL, and Tre-L' s MIC values being 1 mg/mL and 0.5 mg/mL for *E. coli* and *B. subtilis*, respectively. The Mal-L was more effective against *E.coli* while Tre-L was more effective against *B.subtilis.* For 6'-*O*-lauroylmaltose, however, the trend differed in another study: the compound was shown to be more effective to *Bacillus* sp. than *E.coli* (Manuel Ferrer et al., 2005): the inhibition percentage of 93 % and 26 % at the concentrations of 0.8 mg/mL and 4 mg/mL. Studies on the antimicrobial properties of trehalose esters have been quite limited. Marathe et al. (2020) investigated the antibacterial effects of trehalose esters with different chain lengths (C8, C12 and C16) and degrees of substitution (monoester and diester) against a variety of Gram-positive and Gram-negative bacteria. Surprisingly, the trehalose monolaurate did not show any effect on *E.coli* and *B. subtilis*, rather, it was quite effective to *P. aeruginosa* (MIC 0.45 mM). Only the trehalose monocaprylate and dicaprylate were found to be inhibitory (30 mM and 16 mM MIC, respectively) to *E.coli* DH5α, indicating the hydrophobic chain length and bacterial strains all affected the inhibitory effect. From the authors' literature review, the exact reasons for these variations were not investigated in thoroughly. The differences in the bacterial strains, the solubilities and purities of the antimicrobial substances, and the studying methods may account for the different observations. The commercial sucrose ester (HLB 16) showed less efficacy than the in-house synthesized disaccharide esters, as the MIC were not found for both bacteria.

In summary, the in-house synthesized disaccharide monoesters showed a more potent antimicrobial effect than the monosaccharide monoesters. The compounds' solubilities or the interactions of the esters with the bacterial cells may give rise to the phenomenon. The Gal-L and Man-L showed significantly better inhibition effects than the Glu-L. Among the monosaccharide esters, the *E.coli* 12024 was more susceptible to the Gal-L and *B. subtilis* was more susceptible to Man-L. Among the disaccharide esters, *B.subtilis* was more prone to Tre-L, whereas *E.coli* was more susceptible to Mal-L.

## 4 Conclusions

The study elucidated the regioselectivity of five types of sugar esters synthesized by enzyme method and provided the structural-functionality relationship. The acylation positions were either the 6- or 6′- position of the sugar structures. The glycosyl group had a significant impact on the synthesis yield and functionalities. The in-house synthesized emulsifiers were not as effective as the commercial sucrose esters in maintaining the emulsion stability, possibly due to low aqueous solubility and the absence of diesters that can provide larger surface coverage thus offer steric hindrance and viscoelasticity at the oil-water interface. The antimicrobial efficacy of the synthesized galactose-, mannose-, maltose- and trehalose esters were found to be more superior than the commercial ones. Among all the in-house synthesized monosaccharide esters, the mannose monolaurate achieved the best foaming and antimicrobial properties, while the two types of disaccharide esters showed similar emulsion-stabilizing, foaming and antimicrobial properties. Future research should focus on improvement of the synthesis yield and exploration on esters that are more surface active and posses more potent antimicrobial efficacy.

## Funding

This work was supported by Changsha Natural Science Foundation Project kq2502259, Central South University of Forestry and Technology's Talent Introduction Scientific Research Startup Fund Project 2021YJ027, Hunan Provincial Education Department Outstanding Youth Project 21B0254, Key Research Project of Dongting Laboratory No. 2024-DTPY-008, the Science and Technology Innovation Program of Hunan Province Project 2023RC3163 and Hunan Provincial Natural Science Foundation of China Project 2024JJ3053.

## CRediT authorship contribution statement

**Kangzi Ren:** Writing – original draft, Funding acquisition, Formal analysis, Conceptualization. **Guilin Chen:** Methodology, Investigation, Data curation. **Ziyi Zhang:** Data curation, Investigation. **Zhao Long:** Writing – review & editing, Conceptualization. **Bo Zhou:** Funding acquisition, Conceptualization. **Wenfang Han:** Writing – review & editing, Funding acquisition, Conceptualization. **Qinlu Lin:** Project administration, Funding acquisition.

## Declaration of competing interest

The authors declare that they have no known competing financial interests or personal relationships that could have appeared to influence the work reported in this paper.

## Data Availability

Data will be made available on request.

## Appendix A Supplementary data

HMBC for Glu-L and Mal-L, and Emulsion Stability Pictures Image 1
