# Supplementary material for: Enzymatic synthesis of some sugar-lauric acid esters by lipase from Candida antarctica and their functionalities as emulsifiers and antibacterial agents
Source: Food Chem X. 2025 Mar 17;27:102383. doi: 10.1016/j.fochx.2025.102383 (PMC11981728; doi:10.1016/j.fochx.2025.102383)
Supplement: Supplementary file 1 — HMBC for Glu-L and Mal-L, and Emulsion Stability Pictures [file mmc1.docx]

Supplementary Materials


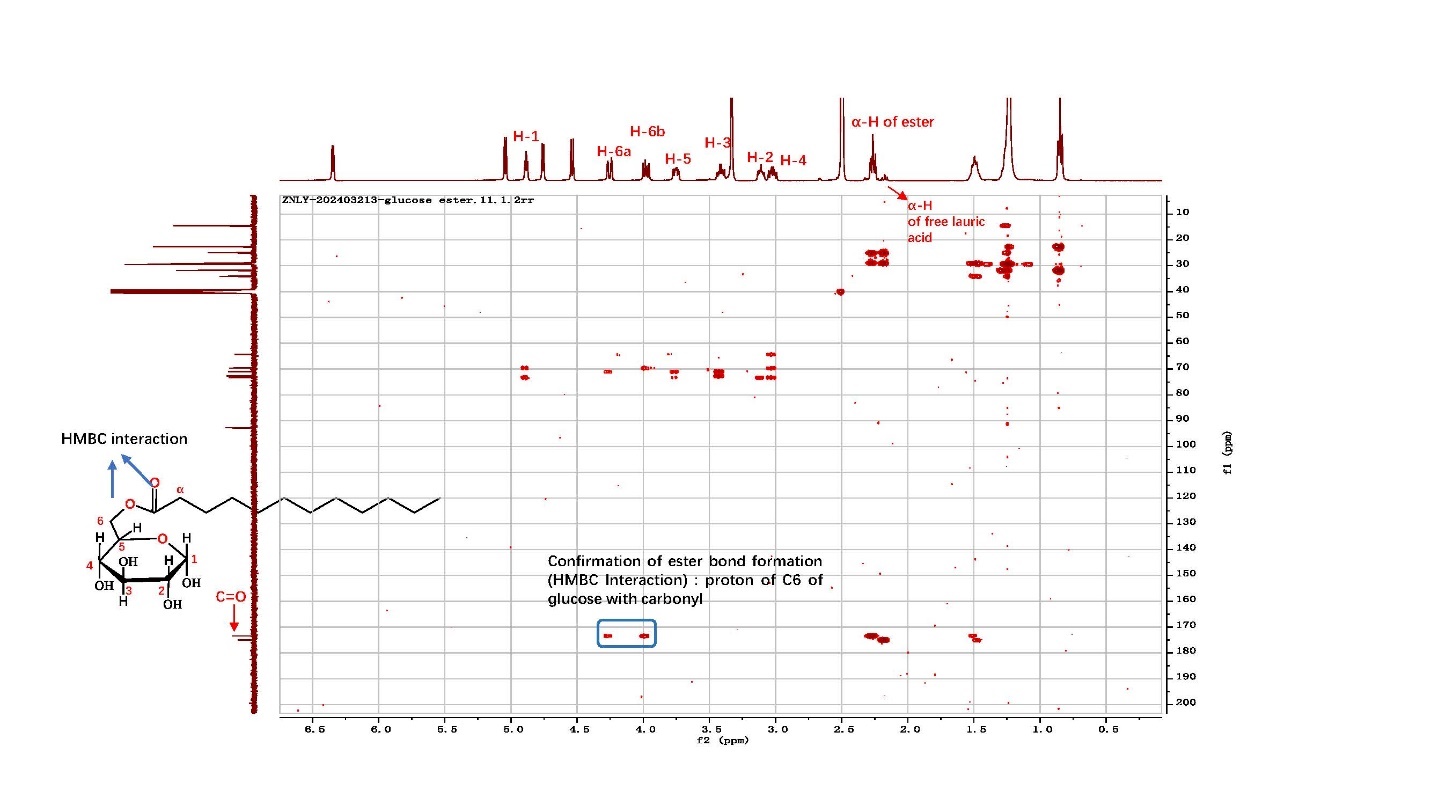


A


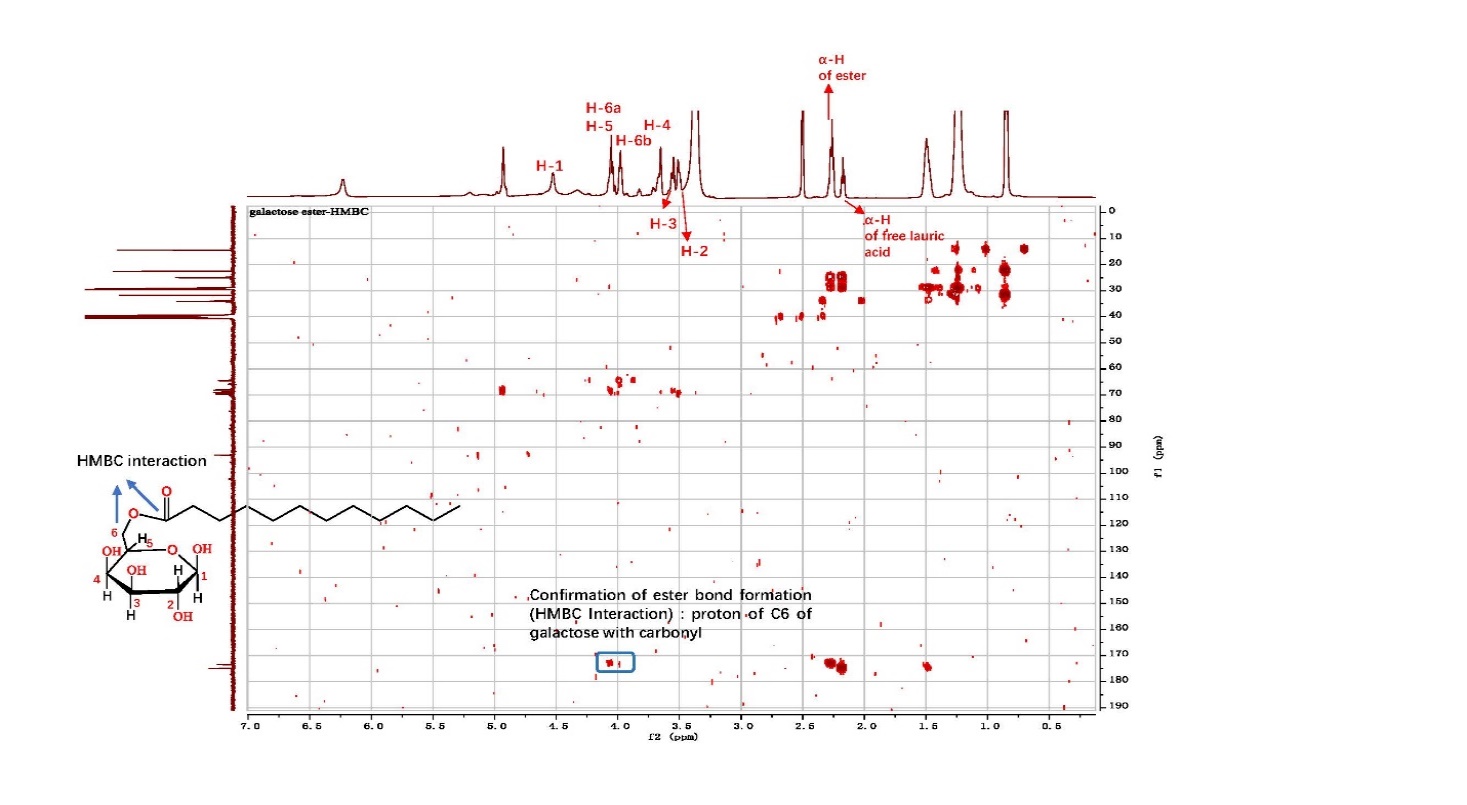


B


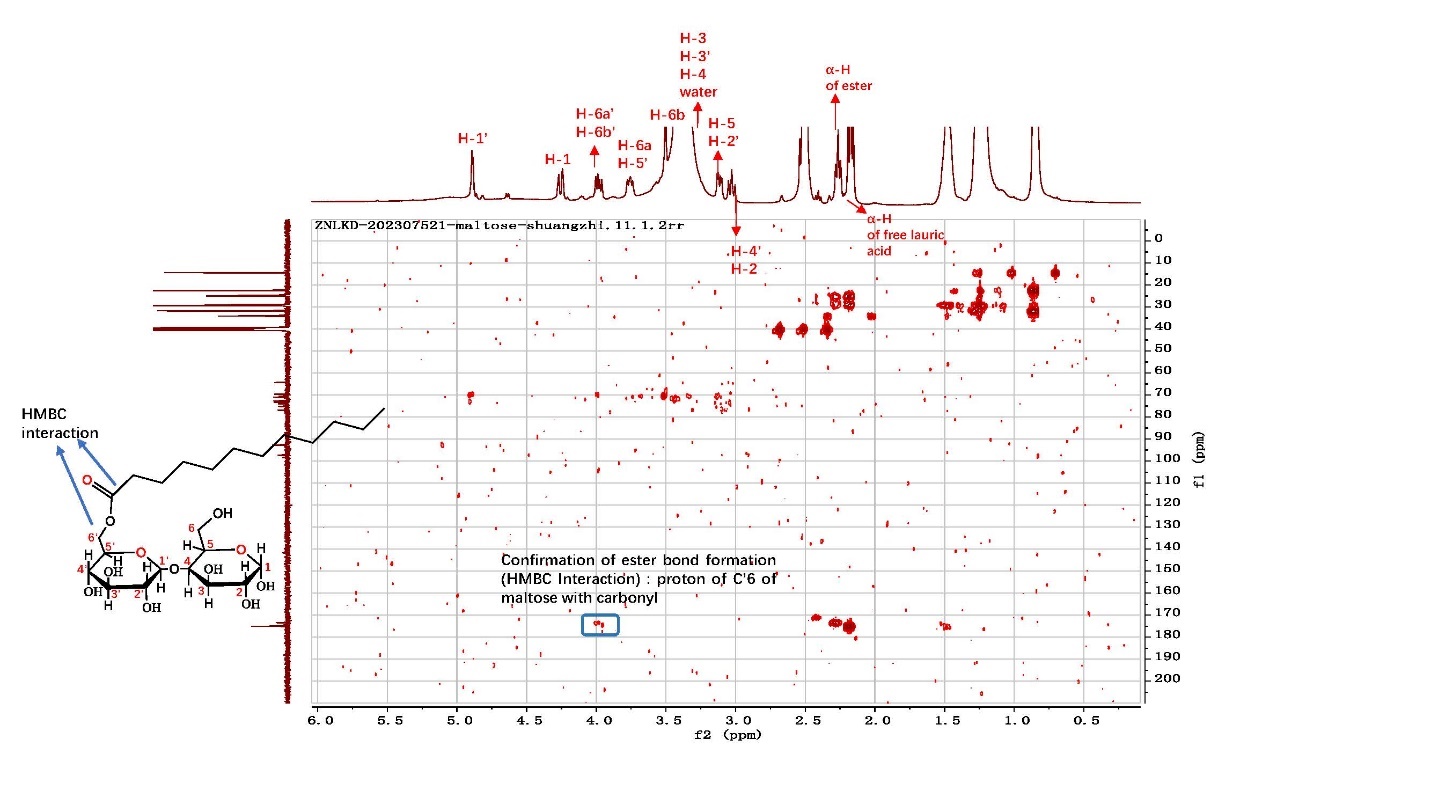


C

Figure. S1. Heteronuclear multiple bond (HMBC) of purified *6-O-lauryl glucose* (A. Glu-L), *6-O-lauryl galactose* (B. Gal-L) and *6’-O-lauryl maltose* (C. Mal-L)


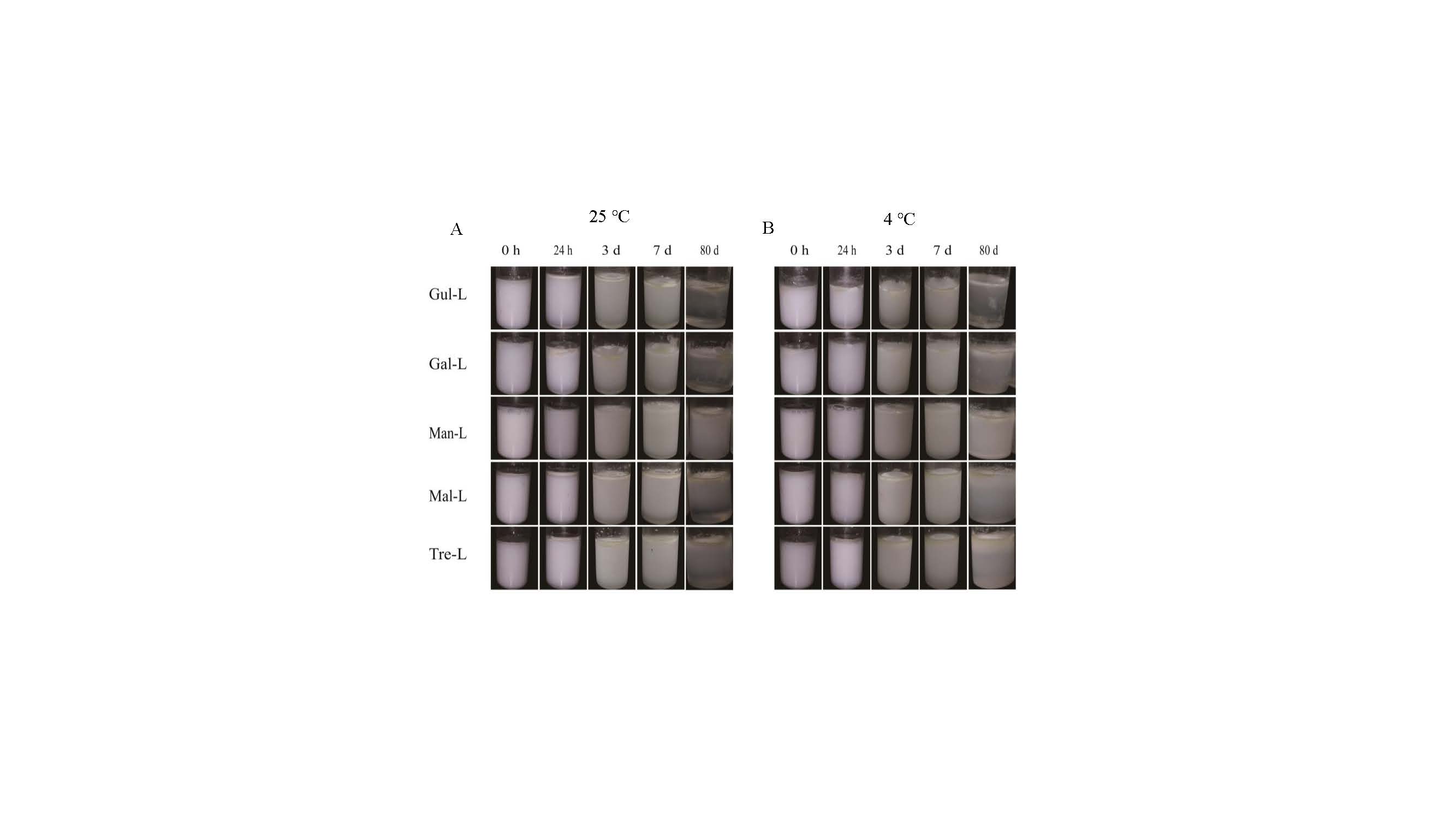


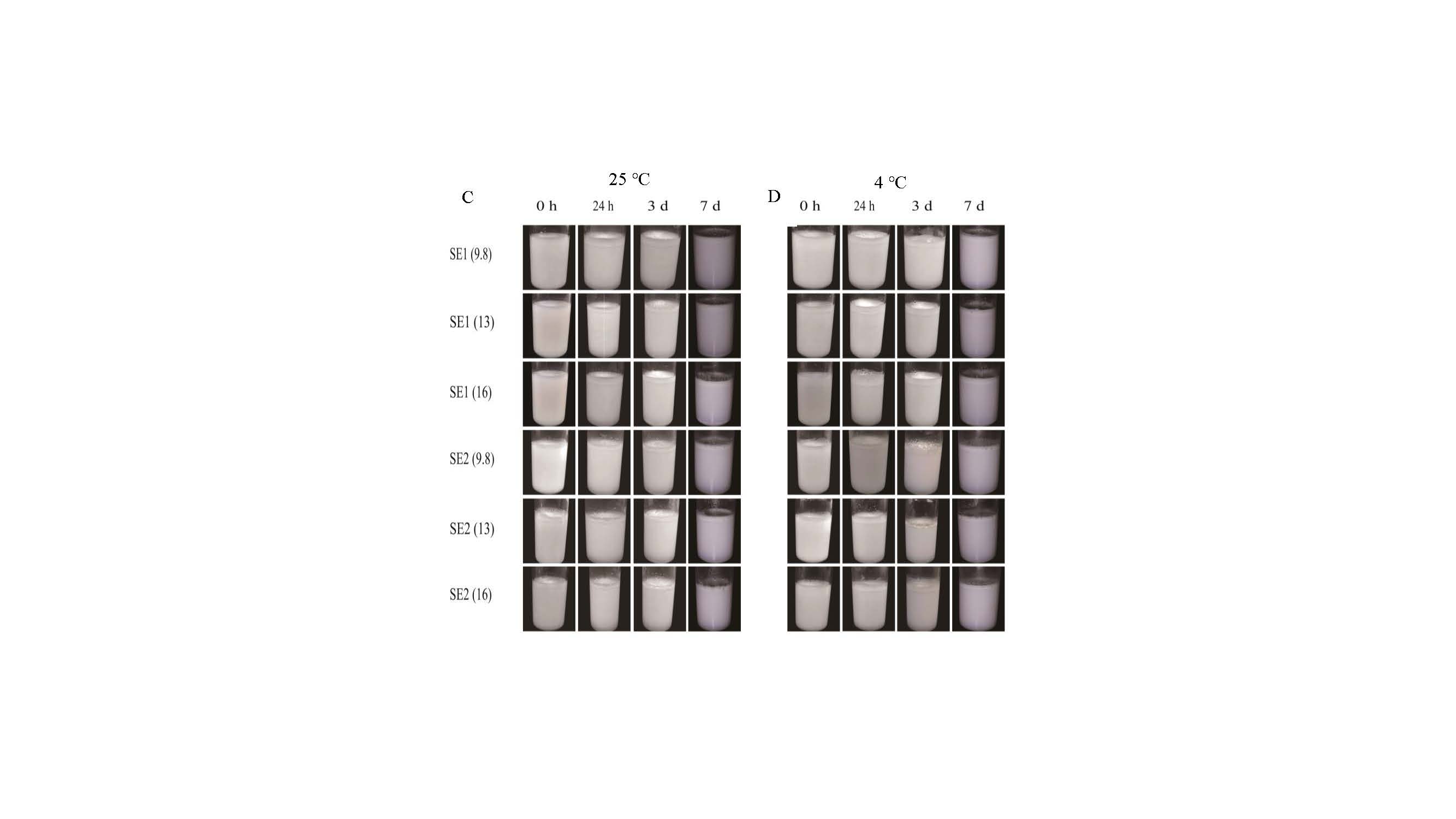
、

Figure. S2. Emulsions stabilized by in-house synthesized sugar esters (A, B) and commercial sucrose esters (C, D) during storage time at 25 ℃ and 4℃.

Glu-L: *6-O-lauryl glucose*; Gal-L*: 6-O-lauryl galactose*; Man-L:*6-O-lauryl mannose*; Mal-L: *6′-O-lauryl maltose*; Tre-L: *6-O-lauryl trehalose* and *6’-O-lauryl trehalose*. SE1-sucrose esters purchased from Mitsubishi Group. SE2-sucrose esters purchased from Liuzhou Aigefu Food Technology Co. The numbers in the bracket indicate the HLB values.
